# Uncommon primary nasosinusal B-cell lymphoma in children: A case report

**DOI:** 10.1016/j.radcr.2024.10.103

**Published:** 2024-11-16

**Authors:** Ihssan Hadj Hsain, Chehrastane Rachida, Lahlou Chaimae, Marrakchi Salma, Boutaleb Joud, Nadia Cherradi, Hafsa El ouazzani, Nazik Allali, Latifa chat, Siham EL haddad

**Affiliations:** Department Of Maternity and Pediatric Radiology HER, Department of Pathology HSR Mohammed V University, Rabat, Morocco

**Keywords:** Lymphoma B, Nasopharyngeal

## Abstract

This review presents the case of a 9-year-old patient who reported headaches and unilateral nasal obstruction, without fever, and with a generally preserved condition. An endoscopic examination revealed a polypoid fleshy mass in the left nasal cavity, along with purulent secretions reaching the inferior turbinate. These findings prompted a facial CT scan to assess the nature of the lesion and the extent of infiltration into surrounding structures. A biopsy and histological analysis confirmed the diagnosis of B-cell lymphoma in the nasal sinuses, a rare occurrence in children. The unusual presentation of this case makes it worthy of sharing.

## Introduction

Malignant lymphomas of the nasopharynx or nasal cavity are rare neoplasms. However, among nonepithelial malignant tumors in this region, lymphomas are the most common. Freeman and his colleagues looked at 1467 cases of extranodal lymphomas and found nasopharyngeal lymphoma in 37 of them and lymphoma affecting the nose and structures nearby in 33 of them [1]. Tumors in the head and neck region are uncommon in children [2]. Lymphoma is the most frequent tumor in this region in pediatric patients, followed by rhabdomyosarcoma, nasopharyngeal carcinoma, thyroid carcinoma, and eosinophilic granuloma [3]. It is also the leading cause of primary malignant disease in the head and neck among children. We present the case of a 9-year-old child with B-cell lymphoma in the sinonasal region in this review.

## Case report

A 9-year-old patient presented with a headache and unilateral nasal obstruction, without a fever, and with a preserved general condition. Clinical examination showed the patient to be in fairly good overall health, afebrile, with no facial deformity. An endoscopic examination showed a polypoid, fleshy mass in the left nasal cavity. It had reached the inferior turbinate after secretions of pus had been aspirated. The mass bled easily upon contact, with lysis observed in the middle and superior turbinates. The rest of the clinical examination was unremarkable, particularly in the lymph node regions.

**Fig. 1:**
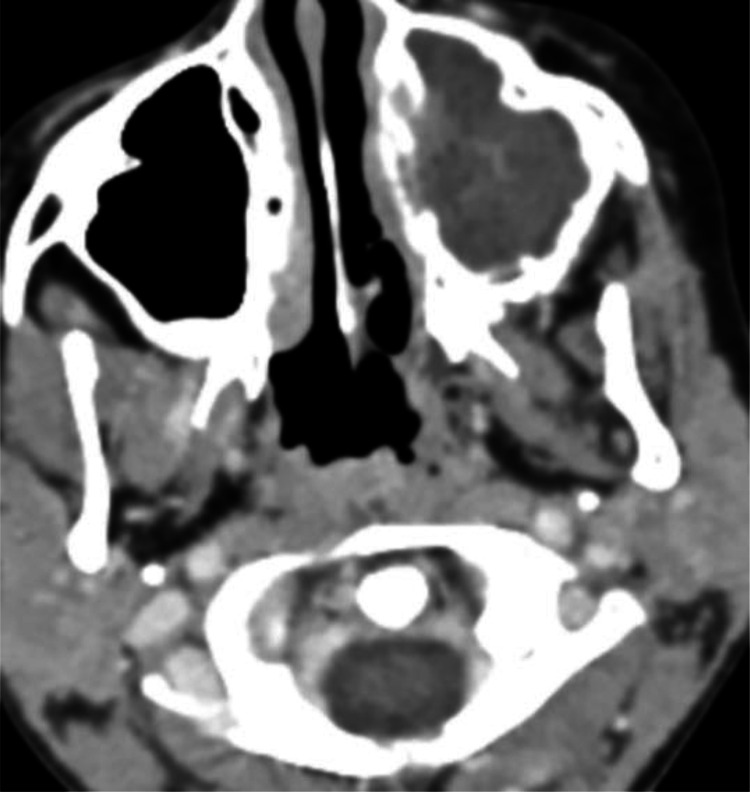
The axial section of a facial CT scan reveals a heterogeneous tissue lesion centered on the left maxillary sinus.

**Fig. 2:**
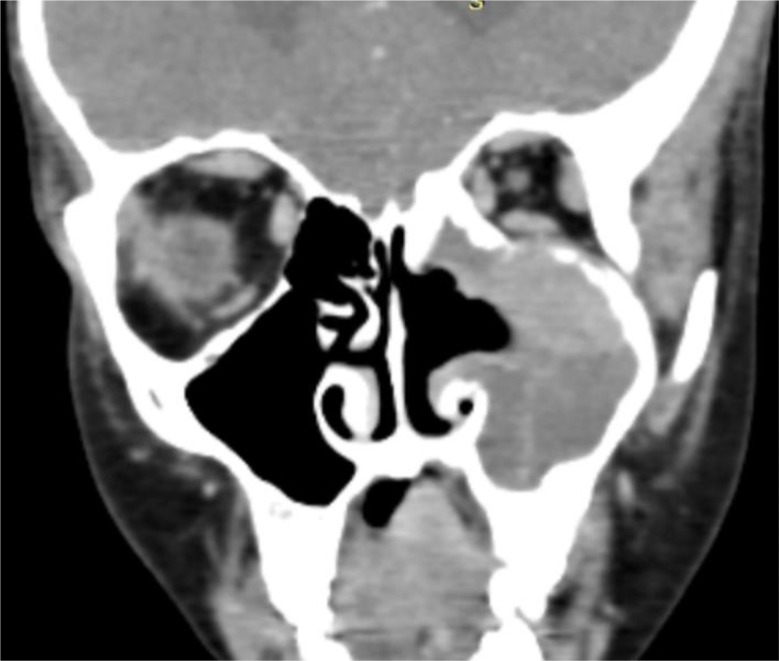
The coronal section of the facial CT scan shows a heterogeneous tissue lesion centered on the left maxillary sinus with lysis of the medial wall and invasion of the lower nasal turbinate.

**Fig. 3:**
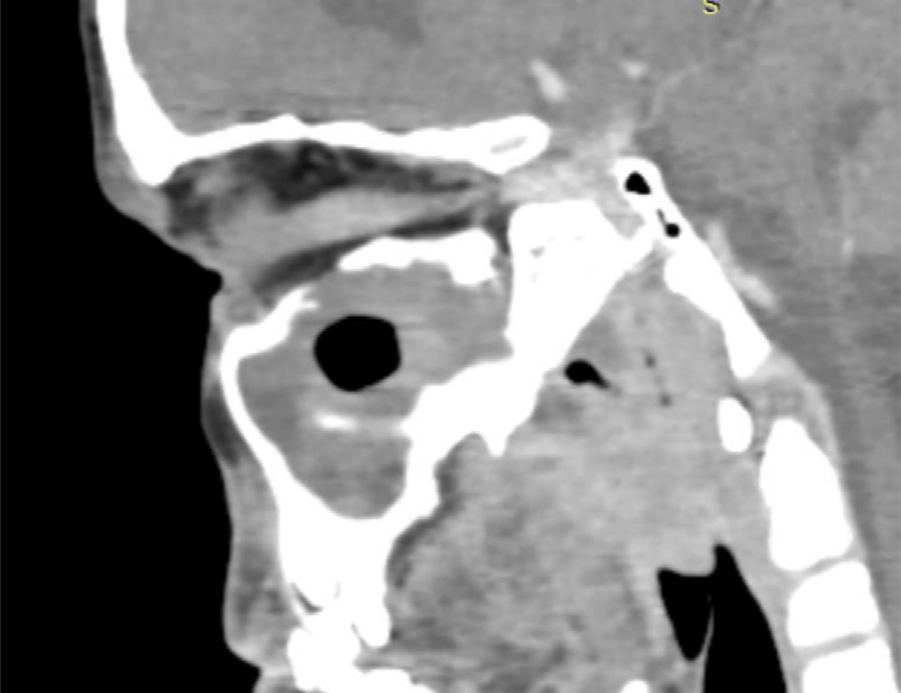
The facial CT sagittal section shows a heterogeneous tissue lesion centered on the left maxillary sinus with lysis of the upper wall and invasion of extraconal fat.

A facial CT scan was performed, revealing a central lesion filling the left maxillary sinus with irregular contours and heterogeneous enhancement after contrast injection (Fig. 1). Medially, the lesion eroded the sinus wall and invaded the ipsilateral inferior turbinate without obstructing the nasal cavity (Fig. 2). Laterally, it caused lysis in areas of the lateral wall. Superiorly, it invaded the ipsilateral ethmoidal cells, eroded the superior wall, infiltrated the extraconal fat, and came into contact with the inferior rectus muscle (Fig. 3).

**Fig. 4:**
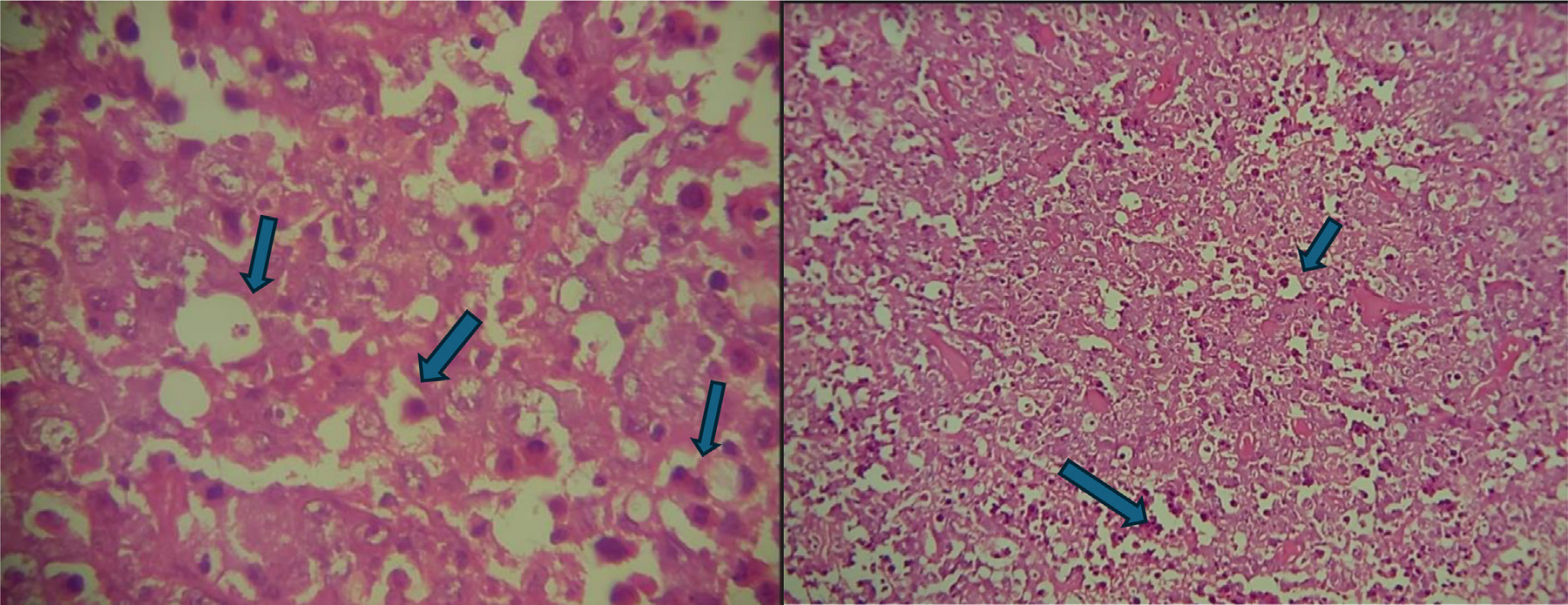
The tumor proliferation in diffuse architecture is composed of large, isolated cells with rounded nuclei and precise limits; the cytoplasm is abundantly eosinophilic and contains some mitotic figures (arrows).

**Fig. 5:**
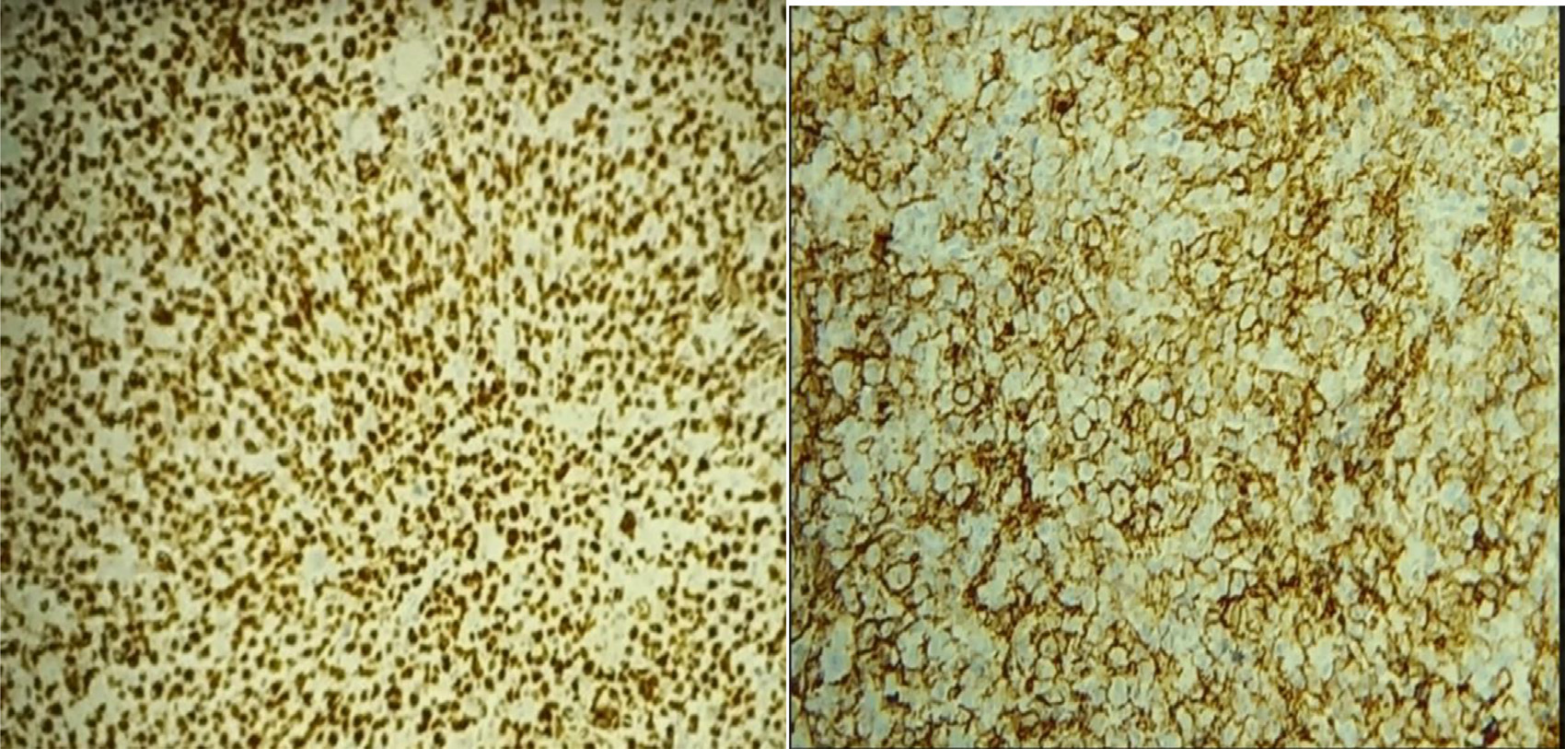
The process involves the diffuse labeling of tumor cells with PAX5 and CD20 receptors.

A biopsy of the friable mass in the left nasal cavity was performed, and histopathological examination revealed a clearly malignant tumor. The tumor was characterized by a diffuse proliferation of isolated large cells with round nuclei and well-defined boundaries, abundant eosinophilic cytoplasm, and some mitotic figures (Fig. 4). Immunohistochemical analysis showed diffuse positive staining for PAX5 and CD20 in the tumor cells (Fig. 5).

Once the diagnosis of lymphoma was confirmed, staging investigations were conducted, including neurological and ophthalmological examinations, which were normal. Laboratory tests were also normal, with a negative sternal puncture and a normal thoraco-abdomino-pelvic CT scan. The lymphoma was classified as stage IE according to the Ann Arbor classification. The patient received 5 cycles of adjuvant chemotherapy following the CHOP protocol, consisting of Adriamycin, Vincristine, Cyclophosphamide, and Prednisone. The patient is currently undergoing treatment, and a follow-up CT scan has not yet been scheduled.

## Discussion

Hodgkin or non-Hodgkin types are the classical classifications for lymphomas. Hodgkin lymphoma follows a bimodal incidence pattern, with the first peak in adolescence and the second in middle age. Conversely, non-Hodgkin lymphoma (NHL) occurs more frequently in the head and neck region in the pediatric population compared to Hodgkin lymphoma [2,4]. Extranodal lymphomas account for 10% to 58% of NHL cases [5]. Primary lymphomas of the paranasal sinuses are rare, representing only 3% of head and neck neoplasms [5], with sinonasal involvement being particularly uncommon, constituting only 0.17% of all lymphomas [6]. B-cell lymphoma is the most common histological type in these cases, while T-cell lymphoma, as in our patient, is exceptionally rare.

Clinically, nasosinusal lymphomas do not present with specific signs, and symptom severity often does not correlate with the tumor size. The clinical presentation varies by histological type. Low-grade lymphomas typically present with a nasal or paranasal mass, causing obstructive symptoms and sometimes lymphadenopathy. Symptoms such as shortness of breath, wheezing, and respiratory sounds are common but not specific to NHL. In contrast, high-grade lymphomas, which account for 38% of NHL in the nasosinusal tract, often present with aggressive symptoms, including nonhealing ulcers, cranial nerve involvement, facial swelling, epistaxis, or pain. High-grade B-cell lymphomas frequently cause soft tissue or bone destruction, particularly in the orbit, leading to exophthalmos, while T-cell lymphomas are associated with perforation or destruction of the nasal septum. Extranodal dissemination is rare, occurring in lymph nodes, skin, and testicles. At presentation, approximately 50% of patients have associated nodal disease, while only 20% exhibit systemic (B) symptoms. Extranodal relapse outside the gastrointestinal tract occurs in 15% of cases, affecting sites such as the larynx, skin, liver, kidneys, and testicles [7].

Radiological imaging is essential for assessing tumor extension, bone destruction, mucosal thickening, and for informing treatment decisions. On CT, nasosinusal lymphomas often appear as irregular, polycyclic tissue masses with erosion or decalcification of the bony walls. Unlike infections or inflammatory conditions, lymphomas typically invade surrounding structures without early bone destruction, which helps differentiate them from other masses like carcinomas. However, in advanced stages, progressive bone erosion may occur. This pattern was observed in our patient, who had a large tumor with relatively subtle bone destruction. MRI is also useful for evaluating tumor extension and distinguishing tumors from inflammatory lesions [8].

The gold standard for diagnosis is histopathological examination of tissue biopsies. In cases of lymph node hypertrophy, excisional biopsy is preferred, but if the mass is limited to the nasal cavity or peripheral nervous system, a generous tissue biopsy is essential to determine the tumor's histological type. Immunohistochemistry is then used to differentiate between B-cell and T-cell lymphomas [[9], [10]].

The differential diagnosis for nasosinusal lymphomas includes mucoceles, infectious etiologies (such as acute or chronic sinusitis), inflammatory antrochoanal polyps, benign or malignant tumors, and, in children, rhabdomyosarcoma, nasopharyngeal carcinoma, thyroid carcinoma, and eosinophilic granulomas [16].

In adults, treatment for nasosinusal lymphoma primarily involves radiotherapy, with the role of surgery remaining unproven in terms of tumor control or survival. The role of adjuvant chemotherapy is still debated. In children, treatment for recurrent NHL may include high-dose chemotherapy with stem cell transplantation or a combination of chemotherapy and monoclonal antibody therapy [11]. Some ongoing clinical trials are exploring the efficacy of combining therapies such as monoclonal antibodies, chemotherapy, stem cell transplantation, and donor lymphocyte infusion [12].

Prognosis depends on the type and stage of the lymphoma, the number of extranodal sites, central nervous system involvement, and the patient's overall condition. High-grade lymphomas, recurrent disease, or widespread dissemination are associated with poor outcomes [[13], [14]]. Two-thirds of patients achieve remission after initial treatment, but one-third relapse, and of those, three-quarters ultimately die from the disease. Survival rates vary by histological type, with 5-year survival rates around 30% for all types, 55% for diffuse large B-cell lymphoma, 33% for peripheral T-cell lymphoma, and 20% for angiocentric lymphoma [15].

## Conclusion

Primary nasosinusal B-cell lymphoma is a rare pathology, especially in children, and should be considered as a differential diagnosis for other malignant tumors of the sinuses. The symptoms are often atypical, depending on the region involved, and the diagnosis is primarily histological. Treatment typically shows a favorable response to chemotherapy.

## Patient consent

For the case report written informed consent for publication of the case was obtained by the first author of the case report.
